# Land use mix and physical activity in middle-aged and older adults: a longitudinal study examining changes in land use mix in two Dutch cohorts

**DOI:** 10.1186/s12966-021-01083-1

**Published:** 2021-02-15

**Authors:** J. M. Noordzij, M. A. Beenackers, J. Oude Groeniger, E. J. Timmermans, I. Motoc, M. Huisman, F. J. van Lenthe

**Affiliations:** 1 Department of Public Health, Erasmus University Medical Center, P. O. Box 2040, 3000 CA Rotterdam, Zuid-Holland The Netherlands; 2 Department of Public Administration and Sociology, Erasmus University, Rotterdam, The Netherlands; 3 Department of Epidemiology and Data Science, Amsterdam Public Health Research Institute, Amsterdam UMC, Vrije Universiteit Amsterdam, De Boelelaan, 1117 Amsterdam, The Netherlands; 4 Department of Sociology, Faculty of Social Sciences, Vrije Universiteit Amsterdam, Amsterdam, The Netherlands; 5 Department of Human Geography and Spatial Planning, Utrecht University, Utrecht, The Netherlands

## Abstract

**Background:**

With urbanization and aging increasing in coming decades, societies face the challenge of keeping aging populations active. Land use mix (LUM) has been associated with cycling and walking, but whether changes in LUM relate to changes in cycling/walking is less known.

**Objectives:**

Our objective was to study the effect of LUM on cycling/walking in two Dutch aging cohorts using data with 10 years of follow-up.

**Methods:**

Data from 1183 respondents from the Health and Living Conditions of the Population of Eindhoven and Surroundings (GLOBE) study and 918 respondents from the Longitudinal Aging Study Amsterdam (LASA) were linked to LUM in 1000-m sausage network buffers at three time-points. Cycling/walking outcomes were harmonized to include average minutes spent cycling/walking per week. Data was pooled and limited to respondents that did not relocate between follow-up waves. Associations between LUM and cycling/walking were estimated using a Random Effects Within-Between (REWB) model that allows for the estimation of both within and between effects. Sensitivity analyses were performed on smaller (500-m) and larger (1600-m) buffers.

**Results:**

We found evidence of between-individual associations of LUM in 1000-m buffers and walking (β: 11.10, 95% CI: 0.08; 21.12), but no evidence of within-associations in 1000-m buffers. Sensitivity analyses using 500-m buffers showed similar between-associations, but negative within-associations (β: -35.67, 95% CI: − 68.85; − 2.49). We did not find evidence of between-individual associations of LUM in any buffer size and cycling, but did find evidence of negative within-associations between LUM in 1600-m buffers and cycling (β: -7.49, 95% CI: − 14.31; − 0.66).

**Discussion:**

Our study found evidence of positive associations between LUM and average walking time, but also some evidence of negative associations between a change in LUM and cycling/walking. LUM appears to be related to cycling/walking, but the effect of changes in LUM on cycling/walking is unclear.

**Supplementary Information:**

The online version contains supplementary material available at 10.1186/s12966-021-01083-1.

## Introduction

In the coming decades, the global population of older adults is projected to increase substantially [1]. As older age is often associated with physical frailty, sustaining good physical functioning is essential. Physical inactivity has been identified as the fourth leading risk factor for global mortality [2] and increasing physical activity (PA) has been marked as a top-priority intervention to reduce death rates of non-communicable diseases [3]. Regular PA contributes to several beneficial health effects for older adults, such as lower risk of cardiovascular disease, diabetes, and cognitive decline [4]. To promote PA among older adults, it is important to foster residential environments that encourage PA as older adults might be especially susceptible to residential factors that discourage an active lifestyle, due to a decline in overall mobility and comparatively more time spent in the neighborhood [5, 6]. Multiple studies have shown positive associations between PA and measures of urban form, such as urban green spaces, public open spaces, residential density, and land use mix [7–9]. Changes in the built environment, such as increased investment in green spaces and pedestrian and cycling infrastructure, as well as transforming cities towards more compact, mixed-used environments can potentially aid in promoting PA [8, 10]. Furthermore, modification of the built environment for health-related purposes could gain more traction in the coming years as a co-benefit of structural urban changes, such as climate control efforts.

One commonly studied physical-environmental exposure with regards to PA is that of land use mix (LUM). LUM represents how evenly different types of land uses are distributed within a specified area [11]. Mixed-use areas contain a variety of different land uses and are believed to encourage PA because they include a larger number of destinations [12, 13]. A systematic review on the neighborhood environment and active travel in older adults found moderate-to-strong evidence of positive associations between LUM and older adults’ total walking [6], while a recent study from Finland found strong evidence in support of the hypothesis that increasing neighborhood density, mixed land use, and access networks may enhance regular walking and cycling [14]. However, much of the evidence linking varying land uses to PA is cross-sectional, which makes it difficult to establish a causal relationship. Many studies adjust for confounding factors, but it remains unclear which factors should be included. Furthermore, selection bias remains an issue as individuals may choose to live in areas based on lifestyle preferences and socioeconomic factors [15]. A physically active person may deliberately choose to live in a PA friendly area, inflating the possible relation between LUM and PA.

Various methods have been applied to account for these methodological shortcomings, such as adjustments for proxy indicators of preferences, as well as applying fixed effects (FE) models that control for time-invariant characteristics, assuming that they remain stable over time. A few studies to date exist that apply such models to analyze how environmental factors relate to PA, but the results are inconclusive. A study conducted in Brisbane, Australia found that any walking for transport versus no walking for transport was increased in association with LUM, but minutes walking per week was not [12], while a Dutch study found weak evidence of associations between changes in green space areas and changes in walking in middle-aged and older adults, but no evidence for cycling [16]. While FE models provide valuable tools for assessing the effects of temporal changes, they disregard between-individual variability. As the method solely relies on within-individual changes, it might not be the best fit for LUM measures, as it is debatable how much LUM changes over time. The primary alternative – the random effects (RE) model – makes use of between-individual variability, but in turn does not remove the effects of time-invariant causes, and assumes that the unmeasured causes are uncorrelated with measured causes. The latter is often a difficult assumption to make and, if violated, will result in omitted-variable bias [17]. Methods exist that combine elements of both RE and FE models and take “the best of both worlds [17].” These models go by different names, such as random effects between-within models (REWB), Mundlak models, or simply hybrid models, and make use of centering of all individual units around their means [18, 19]. Such models can be of great value for research considering the impact of LUM on PA as they not only explore the differences between individuals, but also how a change in LUM might influence a change in PA. However, these models have only been scarcely applied within the public health domain [19].

Further complicating the evidence in the field of environment-PA research is a lack of consistency in both geographic units and scale used to define the residential environment [20, 21]. To quantify environmental exposures, researchers traditionally relied on neighborhood-level data, such as pre-existing administrative units. A more refined method that is especially relevant for PA comes with the use of network buffers that define buffers as areas accessible via a street network. The “sausage” or “line-based” buffering method selects roads within a certain distance of the individual and creates a buffer around these roads by a set distance (e.g. 25 m). This ensures that only those features that are directly accessible from the street network are selected. This method has the key advantage that it is based directly on the road network where people travel [21, 22]. Sausage buffers therefore offer an attractive alternative to more traditional Euclidian buffers – especially when PA is concerned – as these buffers represent areas that are actually accessible via the road network.

Our study uses sausage buffers to define LUM within the individual’s residential environment and links these data to cycling and walking outcomes. We linked data from two Dutch cohorts with 7 to 10 years of follow-up to a harmonized land use dataset, and explored both within-person and between-person associations of LUM on cycling/walking using a REWB model.

## Methods

### Study population

Data were obtained from two longitudinal cohort studies on aging in the Netherlands that are participating in the MINDMAP project [23]: the Health and Living Conditions of the Population of Eindhoven and Surroundings (GLOBE) study, and the Longitudinal Aging Study Amsterdam (LASA). The GLOBE study is a prospective cohort study on the role of living conditions for health in the Netherlands [24]. The 2004 sample of GLOBE participants who resided in the city of Eindhoven and surrounding areas was selected for the analyses (*n* = 4775) with follow-up data collected for the years 2011 and 2014. The LASA study is a longitudinal population-based study of the predictors and consequences of aging in the Netherlands [25]. The 2005/2006 LASA sample of participants who resided in the cities of Amsterdam, Zwolle, and Oss and their surrounding areas was selected for the analyses (*n* = 2165) with follow-up data collected for the years 2008/2009 and 2011/2012. The residential addresses of these respondents were geocoded using geographical software package QGIS [26] and a geocoding plug-in developed by the Dutch National Spatial Data Infrastructure (PDOK) [27]. To maintain respondent privacy, addresses were extracted and geocoded using a process previously described [23, 28]. Respondents whose addresses could not be geocoded, who did not participate in all three data collection waves, or who moved outside of the study area of the respective cohorts were excluded. The sample was limited to respondents that did not relocate during follow-up waves, resulting in a final sample of 1183 respondents aged 26 to 85 for GLOBE and 918 respondents aged 57 to 93 for LASA. Sensitivity analyses were performed on the total sample including respondents that moved between follow-up waves (Supplementary File 1).

### Land use exposure measures

**Fig. 1:**
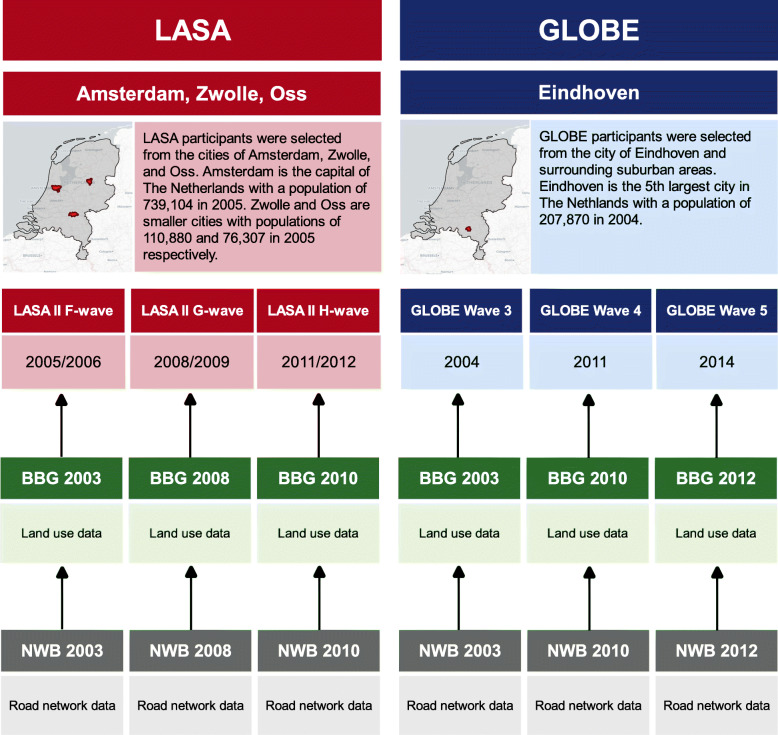
Overview of the land use measures and the cohorts included in this study. Basemap:© Open street map contributors

Exposure measures were obtained using the dataset ‘Bestand Bodemgebruik’ (BBG) which is maintained by Statistics Netherlands [29]. The BBG database is a harmonized dataset based on ‘Top10NL’ digital 1:10,000 topographic maps provided by the Dutch mapping agency Kadaster [30]. The harmonization of the BBG data ensures that observed changes are representative of actual changes in the environment and not related to changes in GIS processing or methodology. The total land use data was grouped into 11 land use categories based on the relevance for cycling and walking. More details on the land use classification can be found in Supplementary File 2. LUM was calculated using network buffers of 1000 m as the main exposure with additional buffers of 500 and 1600 m for sensitivity analyses. The Dutch ‘Nationaal Wegenbestand’ (NWB) database [31] was used for the calculation of the network buffers. The NWB is an open source database with all publicly available roads in the Netherlands with either a street name or a road number. Roads that are not available to pedestrians and cyclists, such as highways, were excluded to provide an accurate estimation of reachable destinations. Sausage buffers were created using line buffers with a radius of 25 m [22, 32]. Land use mix was calculated for all buffer sizes using the following entropy formula:

$$ LUM=-\frac{\left[\sum \limits_{j=1}^N{p}^j\ln \left({p}^j\right)\right]}{\ln (N)} $$

whereby *LUM* is an entropy score with a value between 0 and 1, *p*^*j*^ the percentage of each land use class *j* of the total buffer area, and *N* the total amount of land use classes. The calculated entropy value represents a measure of heterogeneity, whereby 1 represents a perfect mix of land use classes and 0 no mix of classes [33]. *N* was set to 11 LUM classes to avoid measurement bias and to improve comparability of the changes in LUM over time [34]. The LUM entropy score was transformed in the analyses to represent a 10% change in LUM to improve interpretation. Cohort data from each wave was linked to both NWB and BBG data from a preceding year, keeping in line with an appropriate chronology of exposure preceding outcome (Fig. 1). LUM exposure data was calculated for all respondents in the final sample.

### Outcome measures of walking and cycling

Walking and cycling outcomes were assessed using self-reported time spent walking and cycling and defined as average minutes spent walking and cycling per week. GLOBE uses the Short Questionnaire to Assess Health enhancing physical activity (SQUASH) tool, which was created by the Dutch National Institute of Public Health and the Environment to measure habitual physical activity levels in an adult population [35]. In accordance with the SQUASH guidelines, it was assumed that participants who filled-in hours or minutes per week, but omitted ‘days per week,’ had been active for at least 1 day. If the number of days was provided without a corresponding time frequency, the median minutes per day of all respondents was substituted. LASA uses the LASA Physical Activity Questionnaire (LAPAQ), which asks respondent how often and for how long they engaged in various activities, including walking and cycling in the last 2 weeks. LAPAQ has been validated against 7-day physical activity diaries and 7-day pedometer counts in a subsample of LASA participants [36]. A final measure of average minutes per week was computed for both cohorts.

### Covariates

Time-invariant characteristics (as measured at baseline) that were included in the analyses include sex (male, female), and education as measured using the International Standard Classification of Education (lowest = ISCED 0–1, low = ISCED 2, middle = ISCED 3–4, high = ISCED 5–7) [37]. Education was considered to be time-invariant because of the relatively old age of the cohorts. Age, marital status (married/partnership, not married, divorced, widowed), household income (monthly; <€1200, €1200–1800, €1800–2600, >€2600), and employment status (employed, non-employed) were included as relevant time-varying confounders. All time-varying covariates for both studies were measured at all three time points, capturing changes that occurred during follow-up. Missing data on covariates were handled via multiple imputation using the covariates listed above as well as self-rated health (excellent, very good, good, fair, poor), smoking (yes, no), and BMI. Only the covariates education, income, and employment (GLOBE), and income and employment (LASA) had missing values, ranging from 2 to 11% for GLOBE and 5–12% for LASA.

### Statistical analyses

The imputed data of both cohorts was pooled, enabling us to observe more changes in the environment as well as increasing variation in environmental exposure, therefore strengthening both the between- and within-analyses. The analyses were restricted to non-movers to limit selection effects. Sensitivity analyses were performed on data from the separate cohorts as well as on the total sample including those who had moved between data collection waves (Supplementary File 1).

We constructed a random effects within-between (REWB) model to conduct the analyses. This model decomposes the time-varying LUM variable into individual-specific means (between-individual estimates) and deviations from those individual-specific means (within-individual estimates). The estimated between-individual regression coefficient represents how the exposure across all participant-observations is related to the outcome, and the within-individual coefficient represents how variation in exposure around the individual’s mean level is related to the outcomes. In addition, the model can include both time-varying and time-invariant covariates. A random intercept is added to account for the dependence of multiple measurements for each participant. The following model was used for the analyses:

$$ {PA}_{it}={\beta}_0+{\beta}_{1W}\left({x}_{it}-{\overline{x}}_i\right)+{\beta}_{2B}{\overline{x}}_i+{\beta}_3{Z}_i+{\beta}_4{\gamma}_i+\left({v}_i+{\epsilon}_{it}\right) $$

whereby *PA*_*it*_ indicates the PA outcome for individual *i* at time *t*, and *x*_*it*_ is the time-varying land use mix variable. The relationship between *x*_*it*_ and *PA*_*it*_ is decomposed into two parts with *β*_1*W*_ representing the average within effect and *β*_2*B*_ the between effect. *β*_3_ represents the effects of time-invariant measures *Z*_*i*_, and *β*_4_ represents the effects of time-varying measures *γ*_*i*_. *v*_*i*_ is the model’s random effect for individuals I, and *ϵ*_*it*_ are the model’s level-1 residuals. More details on the modeling approach can be found in Supplementary File 3. All analyses were performed using R [38].

## Results

Both cohorts consist of middle-aged and older adults with the mean age ranging from 53 (GLOBE) to 69 years (LASA) at baseline (Table 1). The respondents had an average LUM entropy score of 0.30 (GLOBE) or 0.24 (LASA) on a scale from 0 to 1. Both the average cycling and walking time was higher for GLOBE with 177 min spent cycling per week and 176 min walking compared to 76 min of cycling and 169 min of walking for LASA.

**Table 1:** Description of the baseline study samples for GLOBE and LASA

	GLOBE *n* = 1183	LASA *n* = 918	POOLED *n* = 2101
**EXPOSURE**	**Mean (SD)**	**Mean (SD)**	**Mean (SD)**
Land use mix in 1000-m buffers, entropy score	0.30 (0.06)	0.24 (0.09)	0.30 (0.07)
**OUTCOMES**			
Average cycling time per week, minutes	177 (240)	76 (111)	133 (201)
Average walking time per week, minutes	176 (248)	169 (226)	173 (239)
**INDIVIDUAL CHARACTERISTICS**			
**Time-invariant characteristics**			
Male, %	48%	44%	46%
Education, %			
Lower secondary or less (ISCED 0–2)	21%	44%	31%
Upper secondary (ISCED 3)	19%	16%	18%
Post-secondary non-tertiary education or short-cycle tertiary education (ISCED 4,5)	25%	19%	22%
Bachelor, master, doctoral, or equivalent (ISCED 6,7,8)	35%	21%	29%
**Time-varying characteristics**			
Age, mean (SD)	56 (12)	68 (8)	60 (12)
Employment status, %			
Currently in paid employment	51%	21%	39%
Currently not in paid employment	49%	79%	61%
Income, %			
< €1200	8%	17%	12%
€1200 - €1800	24%	32%	27%
€1800 - €2600	32%	51%	40%
> €2600	36%	n.a.*	21%*
Marital status, %			
Married or registered partnership	80%	69%	75%
Never married	9%	6%	8%
Divorced	6%	6%	6%
Widowed	5%	19%	11%

* The highest income class for LASA consists of respondents with an income of > €2270

Within-individual changes in LUM were observed for approximately 44% of all person-observations (Table 2). The observed changes consisted of both decreases and increases in the LUM which corresponded to an average 5% decrease and an average 3% increase. Within-individual changes were also observed for both outcomes with approximately 18% (cycling) and 14% (walking) reporting no change in the average amount of minutes spent walking/cycling per week.

**Table 2:** Within-individual changes in land use mix in 1000-m buffers and average cycling and walking time per week between 2004 and 2014 using pooled data from respondents that did not relocate during follow-up

	Decrease		No Change		Increase	
n = 6303 person-observations	Mean	n	Mean	n	Mean	n
**Exposure**						
Land use mix in 1000-m buffers	−0.05	942	0	3513	0.03	1848
**Outcomes**						
Average cycling time per week (minutes)	−120	2974	0	1157	159	2172
Average walking time per week (minutes)	− 182	2635	0	905	180	2763

REWB models provided no evidence of within or between associations between LUM in 1000-m buffers and the average time spent cycling (Table 3). Sensitivity analyses conducted on 1600-m buffers provided no evidence of between-associations, but did provide evidence of a negative association between a within-individual change in LUM and average time spent cycling (β: -7.49, 95% CI: − 14.31; − 0.66) (Supplementary File 1, Table 5). These results suggest that a 10% change in LUM in 1600-m buffers is associated with a decrease in cycling time per week of 7.49 min.

**Table 3:** Within and between associations of land use mix in 1000-m buffers and average minutes cycling and walking per week using pooled data on respondents that did not relocate during follow-up

*n* = 6303 person observations	**WITHIN EFFECTS**		
**REWB model**^a^	β	95% CI	*p*-value
Land use mix in 1000-m buffers			
Average cycling time per week (minutes)	−5.55	−17.17; 6.07	0.349
Average walking time per week (minutes)	0.75	−14.31; 15.80	0.922
	**BETWEEN EFFECTS**		
REWB model*	β	95% CI	*p*-value
Land use mix in 1000-m buffers			
Average cycling time per week (minutes)	5.06	−4.91; 15.04	0.320
Average walking time per week (minutes)	11.10	0.08; 22.12	0.048

^a^adjusted for study, time-invariant individual characteristics sex and education, and time-varying characteristics age, employment, income, and marital status

REWB models modelling the average time walking showed evidence of positive between-individual associations between average LUM in 1000-m buffers and the average walking time (β: 11.10, 95% CI: 0.08; 21.12), indicating that a 10% change in LUM in 1000-m buffers is associated with an increase of minutes walked per week of 11.10 min. Sensitivity analyses conducted using 500-m buffers showed similar between-individual associations, but also negative within-individual associations (β: -35.67, 95% CI: − 68.85; − 2.49) (Supplementary File 1, Table 9), suggesting that a 10% change in LUM in 500-m buffers is negatively associated with average time spent walking per week.

## Discussion

In the present study, we found evidence of between-individual associations of land use mix in 1000-m buffers and the average walking time per week. We also found comparable between-associations in the smaller 500-m buffers, adding to the robustness of these results. We did not find evidence of within-individual associations between LUM in 1000-m buffers and walking nor did we find evidence of within- or between-individual associations between LUM in 1000-m buffers and cycling. We did find evidence of a negative within-effect on cycling in larger 1600-m buffers, and evidence of a negative within-effect on walking in 500-m buffers.

The 1000-m network buffer is a commonly used exposure measure in PA research as it is believed to be a reasonable distance that people can walk [12]. The associations that we found for this buffer are in line with other studies on this subject. For example, a recent study using the GLOBE data found no evidence of within-associations of green spaces in 1000-m buffers on cycling and walking outcomes [16]. Our study also found no evidence of within-associations between a change in LUM in 1000-m buffers and cycling/walking. These findings raise questions if the observed changes in the 1000-m buffers are large enough to observe a change in cycling/walking. A recent study conducted in Eindhoven, The Netherlands that used similar environmental exposures in 1000-m buffers concluded that it did not find evidence for a change in green space exposure being related to a change in mental health [39]. This study did find some evidence of cross-sectional between-individual associations, and argued that there may have been too few observed changes in the environmental exposure in 1000-m buffers. A study conducted in Brisbane, Australia in adults aged 40 to 60 found that results of estimates from random effects models indicated positive associations between any walking for transport and an increase in LUM of 10%, which is in line with the between-associations that we observed for walking [12]. This Australian study also found positive, if less pronounced, within-individual associations. While our study did not observe within-associations for our main exposure buffers, we did observe within-associations for the smaller 500-m buffers, but these were the inverse of the between associations.

Several issues may contribute to the explanation of the negative within-individual associations in our sensitivity analyses. It is important to note that little consensus exists about what buffer sizes to use when analyzing how LUM and cycling/walking relate, with other studies reporting both smaller and larger buffers [40]. Furthermore, a recent systematic review on the physical environment and active travel in older adults concluded that not much is known about the optimal mix and number of destination types that might promote active travel in this age group [6]. Several studies have concluded that associations between environmental exposures and health outcomes can vary greatly based on the size and type of the buffers used (“crow-fly” Euclidian buffers or network buffers) [21]. Some explanation might therefore be found in the definition of our exposure measures. A study conducted in the Netherlands among older adults found a mean distance of 1997 m for cycling trips and 1101 m for walking trips [41]. As both the GLOBE and LASA cohorts include a large proportion of older adults, we included a larger buffers of 1600 m (one mile) in our sensitivity analyses. The 1600-m buffer is another commonly used buffer and can be especially relevant for cycling as larger distances can be covered compared to walking. We also included a smaller buffer of 500 m in our sensitivity analyses to test whether LUM in this smaller buffer was associated with walking. This is especially important in a population of primarily older adults as their physical functioning might deteriorate over time, confining their PA to a smaller area. However, the results for the larger and smaller buffer sizes were contrary to what we expected based on the existing literature. For example, a study conducted in Perth, Australia in middle-aged adults found that an increase in access to destinations in the residential environment was associated with taking-up cycling, providing evidence that changes in the built environment may support the uptake of cycling among formerly non-cycling adults [42]. Our study did not find evidence that a change in LUM in the residential environment is associated with time spent cycling in our main exposure buffers of 1000 m and some evidence of negative associations between LUM and cycling in larger 1600-m buffers (Supplementary File 1, Table 5). Explanations for these results may be found in age differences between the studies, cultural differences between cycling in The Netherlands and Australia, but also in the definition of the exposure and the mechanisms between LUM and cycling outcomes. Whereas the study in Perth included respondents that moved to a new residential neighborhood, our study specifically only included respondents that did not relocate during follow-up. The within-changes are therefore indicative of changes in the residential environment and not the result of moving to a different residential environment. Different mechanisms may therefore be at play when compared to the effect that moving to a different neighborhood can have. As our study provides mixed results, more research is needed that explores how changes in the residential environment relate to cycling/walking. This is not only an important question from a scientific point of view, but also from a policy perspective as it provides policy makers with more insights how a change in the environment might relate to a change in cycling/walking. More longitudinal research on this topic is therefore urgently needed; a call that has been echoed by other authors in the field in recent years [43].

### Strengths & limitations

The present study adds to the literature on how the residential environment relates to cycling and walking by using data from two Dutch cohorts with 10 years of follow-up and linking this data to harmonized LUM exposures. By pooling data from two Dutch cohorts, we were able to both increase variation in environmental exposures as well as increase the statistical power of our analyses. Our study provides more evidence on how LUM and cycling/walking relate, by considering the effects of changes in LUM on cycling/walking in a Dutch socio-spatial context where cycling is a big part of everyday life, and for cities that are already very compact compared to those in other countries such as Australia or the United States. Evidence from such countries suggests that a move towards more compact cities with a mixed-use environment can have a positive effect on cycling and walking, but there is little evidence from cities that are already very compact and dense such as the ones in this study [13].

Our study also fills an important methodological gap by exploring both between-individual and within-individual associations of LUM on cycling/walking. By applying the REWB framework to longitudinal data of respondents that did not relocate during follow-up, we gain more insight into how different levels of LUM affect cycling/walking and how a change in LUM can potentially influence the average cycling and walking time. The REWB model retains the advantages of the standard FE model, but also incorporates between-individual variation, while allowing to control for measured time-invariant confounders. By retaining the virtues of the standard FE approach, it helps to infer potential causal relationships between changes in LUM and cycling/walking that have more potential for evidence-based action [19]. It also helps to answer a relevant (policy) question: is a change in LUM in the residential environment associated with a change in cycling/walking? As most of the research on LUM and cycling/walking is cross-sectional, answering this question can broaden the understanding of potential causal pathways between LUM and PA.

The use of sausage network buffers offers numerous improvements over more traditional Euclidian or “crow-fly” buffers that do not consider if the street network allows or prevents access to specific locations. A study comparing different buffer types for PA research concluded that the sausage buffer method remains the most defensible method for creating network buffers as it increases both comparability and repeatability [21]. By including multiple individual-specific network buffers and by excluding roads that are not accessible to pedestrians and cyclists, we aimed to provide an accurate exposure measure that ensures that only those features that are accessible from the road network are included. By applying the buffers to a harmonized land use dataset, we ensured that changes observed in the data are representative of actual changes in the environment and not the result of changes in data processing of GIS methodology.

Our study also has some limitations to consider. First, while individual-level network buffers offer great improvements in measuring exposure compared to more traditional neighborhoods, we were not able to control for other urban-environmental and social-urban factors, such as residential density, safety, or neighborhood socio-economic status. A study conducted in Amsterdam, The Netherlands found evidence that neighborhood safety was associated with cycling [44]. As we used individual-specific network buffers, we were not able to control for such effects in our analyses. Secondly, we were also not able to control for time spent away from the residential environment. However, it has been theorized that older adults may be particularly susceptible to environmental factors in the residential environment as they are likely to spend more time closer to home than younger adults [5]. Thirdly, all cohort waves are separated by 3 years with the exception of GLOBE waves 3 and 4, which are separated by 7 years (Fig. 1). This longer follow-up period could potentially influence physical functioning and cycling/walking time. As our study population has a large proportion of older adults, decay of physical functioning during follow-up could negatively impact cycling and walking time, possibly influencing the within-individuals estimates. Finally, in order to pool the data from both cohorts, variables had to be retrospectively harmonized, which means that study variables are harmonized after they have been collected. While retrospective harmonization is a good way to make comparisons between cohorts possible, it does inherently come with the limitation that some detail is lost in the process. For example, income classes in both cohorts did not match well and therefore had to be generalized in order to be comparable. Harmonization choices like these inevitably lead to a loss in sensitivity and specificity of the data. More prospective harmonization would alleviate these limitations and therefore make better comparisons between cohorts possible.

## Conclusions

The present study found evidence of between-individual associations of land use mix in the residential environment and the average walking time per week, as well as some evidence of negative within-associations between land use mix and the average cycling/walking time in respondents that did not move to a different residential address during follow-up. These findings advocate the use of research methods that combine both between- and within-individual analyses in order to gain more understanding of how land use mix in the residential environment can relate to cycling/walking. More longitudinal research is needed to explore how changes in land use mix over time can influence cycling and walking outcomes.

## Supplementary Information

**Additional file 1.**

## Data Availability

The datasets generated for the MINDMAP project are not publicly available due to study participant privacy considerations. However, data access can be requested from the individual cohort studies via the respective data access procedures in place. The BBG exposure data is openly accessible via Statistics Netherlands.
