# Supplementary material for: Land use mix and physical activity in middle-aged and older adults: a longitudinal study examining changes in land use mix in two Dutch cohorts
Source: Int J Behav Nutr Phys Act. 2021 Feb 15;18:29. doi: 10.1186/s12966-021-01083-1 (PMC7885364; doi:10.1186/s12966-021-01083-1)
Supplement: Supplementary file 1 — Additional file 1. [file 12966_2021_1083_MOESM1_ESM.docx]

# **SUPPLEMENTARY FILES**

**SUPPLEMENTARY FILE 1 – SENSITIVITY ANALYSES**

**INDIVIDUAL COHORTS**

**Supplementary table 1: Within and between associations of land use mix in 1000-meter buffers and average minutes cycling and walking per week for the total GLOBE cohort**

| n = 4,645 person observations | **WITHIN EFFECTS** | | |
| --- | --- | --- | --- |
| **REWB model*** | β | 95% CI | p-value |
| Land use mix in 1000-meter buffers  Average cycling time per week (minutes)  Average walking time per week (minutes) | -6.42  -1.57 | -20.75 ; 7.92  -16.68 ; 13.53 | 0.380  0.838 |
|  | **BETWEEN EFFECTS** | | |
| **REWB model*** | β | 95% CI | p-value |
| Land use mix in 1000-meter buffers  Average cycling time per week (minutes)  Average walking time per week (minutes) | 7.41  10.13 | -9.70 ; 24.52  -5.25 ; 25.51 | 0.396  0.197 |

*adjusted for time-invariant individual characteristics gender and education, and time-varying characteristics age, employment, income, and marital status.

**Supplementary table 2: Within and between associations of land use mix in 1000-meter buffers and average minutes cycling and walking per week for the total LASA cohort**

| n = 3,342 person observations | **WITHIN EFFECTS** | | |
| --- | --- | --- | --- |
| **REWB model*** | β | 95% CI | p-value |
| Land use mix in 1000-meter buffers  Average cycling time per week (minutes)  Average walking time per week (minutes) | -0.40  8.81 | -10.88 ; 10.07  -8.46 ; 26.08 | 0.940  0.317 |
|  | **BETWEEN EFFECTS** | | |
| **REWB model*** | β | 95% CI | p-value |
| Land use mix in 1000-meter buffers  Average cycling time per week (minutes)  Average walking time per week (minutes) | 3.85  15.74 | -1.98 ; 9.68  4.07 ; 27.42 | 0.196  0.008 |

*adjusted for time-invariant individual characteristics gender and education, and time-varying characteristics age, employment, income, and marital status.

**TOTAL SAMPLE INCLUDING MOVERS**

**Supplementary table 3: Within and between associations of land use mix in 1000-meter buffers and average minutes cycling and walking per week using the total sample of pooled data**

| n = 7,998 person observations | **WITHIN EFFECTS** | | |
| --- | --- | --- | --- |
| **REWB model*** | β | 95% CI | p-value |
| Land use mix in 1000-meter buffers  Average cycling time per week (minutes)  Average walking time per week (minutes) | -2.62  2.15 | -12.12 ; 6.88  -9.26 ; 13.55 | 0.589  0.712 |
|  | **BETWEEN EFFECTS** | | |
| **REWB model*** | β | 95% CI | p-value |
| Land use mix in 1000-meter buffers  Average cycling time per week (minutes)  Average walking time per week (minutes) | 5.18  13.80 | -3.55 ; 13.90  4.37 ; 23.23 | 0.245  0.004 |

*adjusted for study, time-invariant individual characteristics gender and education, and time-varying characteristics age, employment, income, and marital status.

**Supplementary table 4: Within-individual changes in land use mix in 500- and 1600-meter buffers using pooled data from respondents that did not relocate during follow-up**

|  | **Decrease** | | **No Change** | | **Increase** | |
| --- | --- | --- | --- | --- | --- | --- |
| n = 6,303 person-observations | Mean | N | Mean | N | Mean | N |
| **Exposure**  Land use mix in 500-meter buffers  Land use mix in 1600-meter buffers | -0.02  -0.01 | 833  860 | 0  0 | 3779  3097 | 0.03  0.05 | 1691  2346 |

**CYCLING – DIFFERENT BUFFER SIZES**

**Supplementary table 5: Within and between associations of land use mix in 1600-meter buffers and average minutes cycling per week using pooled data on respondents that did not relocate during follow-up**

| n = 6,285 person observations | **WITHIN EFFECTS** | | |
| --- | --- | --- | --- |
| **REWB model*** | β | 95% CI | p-value |
| Land use mix in 1600-meter buffers  Average cycling time per week (minutes) | -7.49 | -14.31 ; -0.66 | 0.032 |
|  | **BETWEEN EFFECTS** | | |
| **REWB model*** | β | 95% CI | p-value |
| Land use mix in 1600-meter buffers  Average cycling time per week (minutes) | 3.57 | -5.41 ; 12.55 | 0.436 |

*adjusted for study, time-invariant individual characteristics gender and education, and time-varying characteristics age, employment, income, and marital status.

**Supplementary table 6: Within and between associations of land use mix in 1600-meter buffers and average minutes cycling per week using the total sample of pooled data**

| n = 7,998 person observations | **WITHIN EFFECTS** | | |
| --- | --- | --- | --- |
| **REWB model*** | β | 95% CI | p-value |
| Land use mix in 1600-meter buffers  Average cycling time per week (minutes) | -2.12 | -8.18 ; 3.94 | 0.493 |
|  | **BETWEEN EFFECTS** | | |
| **REWB model*** | β | 95% CI | p-value |
| Land use mix in 1600-meter buffers  Average cycling time per week (minutes) | 4.30 | -4.63 ; 12.23 | 0.288 |

*adjusted for study, time-invariant individual characteristics gender and education, and time-varying characteristics age, employment, income, and marital status.

**Supplementary table 7: Within and between associations of land use mix in 1600-meter buffers and average minutes cycling per week for respondents that did not relocate during follow-up for the GLOBE cohort**

| n = 3,531 person observations | **WITHIN EFFECTS** | | |
| --- | --- | --- | --- |
| **REWB model*** | β | 95% CI | p-value |
| Land use mix in 1600-meter buffers  Average cycling time per week (minutes) | -6.89 | -16.41 ; 2.62 | 0.155 |
|  | **BETWEEN EFFECTS** | | |
| **REWB model*** | β | 95% CI | p-value |
| Land use mix in 1600-meter buffers  Average cycling time per week (minutes) | 3.06 | -14.67 ; 20.79 | 0.735 |

*adjusted for study, time-invariant individual characteristics gender and education, and time-varying characteristics age, employment, income, and marital status.

**Supplementary table 8: Within and between associations of land use mix in 1600-meter buffers and average minutes cycling per week for respondents that did not relocate during follow-up for the LASA cohort**

| n = 2,754 person observations | **WITHIN EFFECTS** | | |
| --- | --- | --- | --- |
| **REWB model*** | β | 95% CI | p-value |
| Land use mix in 1600-meter buffers  Average cycling time per week (minutes) | -10.66 | -18.73 ; -2.60 | 0.010 |
|  | **BETWEEN EFFECTS** | | |
| **REWB model*** | β | 95% CI | p-value |
| Land use mix in 1600-meter buffers  Average cycling time per week (minutes) | 2.75 | -2.85 ; 8.35 | 0.336 |

*adjusted for study, time-invariant individual characteristics gender and education, and time-varying characteristics age, employment, income, and marital status.

**WALKING – DIFFERENT BUFFER SIZES**

**Supplementary table 9: Within and between associations of land use mix in 500-meter buffers and average minutes walking per week using pooled data on respondents that did not relocate during follow-up**

| n = 6,285 person observations | **WITHIN EFFECTS** | | |
| --- | --- | --- | --- |
| **REWB model*** | β | 95% CI | p-value |
| Land use mix in 500-meter buffers  Average walking time per week (minutes) | -35.67 | -68.85 ; -2.49 | 0.035 |
|  | **BETWEEN EFFECTS** | | |
| **REWB model*** | β | 95% CI | p-value |
| Land use mix in 500-meter buffers  Average walking time per week (minutes) | 11.39 | -0.28 ; 23.05 | 0.056 |

*adjusted for study, time-invariant individual characteristics gender and education, and time-varying characteristics age, employment, income, and marital status.

**Supplementary table 10: Within and between associations of land use mix in 500-meter buffers and average minutes walking per week using the total sample of pooled data**

| n = 7,998 person observations | **WITHIN EFFECTS** | | |
| --- | --- | --- | --- |
| **REWB model*** | β | 95% CI | p-value |
| Land use mix in 500-meter buffers  Average walking time per week (minutes) | -4.16 | -22.94 ; 14.62 | 0.664 |
|  | **BETWEEN EFFECTS** | | |
| **REWB model*** | β | 95% CI | p-value |
| Land use mix in 500-meter buffers  Average walking time per week (minutes) | 10.90 | 0.89 ; 20.91 | 0.033 |

*adjusted for study, time-invariant individual characteristics gender and education, and time-varying characteristics age, employment, income, and marital status.

**Supplementary table 11: Within and between associations of land use mix in 500-meter buffers and average minutes cycling per week for respondents that did not relocate during follow-up for the GLOBE cohort**

| n = 3,531 person observations | **WITHIN EFFECTS** | | |
| --- | --- | --- | --- |
| **REWB model*** | β | 95% CI | p-value |
| Land use mix in 500-meter buffers  Average walking time per week (minutes) | -28.45 | -72.76 ; 15.86 | 0.208 |
|  | **BETWEEN EFFECTS** | | |
| **REWB model*** | β | 95% CI | p-value |
| Land use mix in 500-meter buffers  Average walking time per week (minutes) | -2.65 | -22.55 ; 16.95 | 0.791 |

*adjusted for study, time-invariant individual characteristics gender and education, and time-varying characteristics age, employment, income, and marital status.

**Supplementary table 12: Within and between associations of land use mix in 500-meter buffers and average minutes cycling per week for respondents that did not relocate during follow-up for the LASA cohort**

| n = 2,754 person observations | **WITHIN EFFECTS** | | |
| --- | --- | --- | --- |
| **REWB model*** | β | 95% CI | p-value |
| Land use mix in 500-meter buffers  Average walking time per week (minutes) | -40.23 | -90.75 ; 10.28 | 0.119 |
|  | **BETWEEN EFFECTS** | | |
| **REWB model*** | β | 95% CI | p-value |
| Land use mix in 500-meter buffers  Average walking time per week (minutes) | 20.66 | 6.54 ; 34.78 | 0.004 |

*adjusted for study, time-invariant individual characteristics gender and education, and time-varying characteristics age, employment, income, and marital status.

# **SUPPLEMENTARY FILE 2**

# **MORE INFORMATION ON THE BBG DATABASE AND LAND USE CLASSIFICATION**

The TOP10NL dataset is the official, national topographical representation of the Netherlands and is maintained by the Dutch mapping agency ‘Kadaster’. Statistics Netherlands converts this topographical data to land use data and publishes the resulting files as open source GIS data. The ‘Bestand Bodemgebruik’ (BBG) is the collection of these files. The BBG dataset is generally updated every two to four years depending on funding sources and research needs. The most recent dataset is distributed by Statistics Netherlands and is reposited in the Dutch National Georegister. Historical files are distributed by the Netherlands institute for permanent access to digital research resources. These files are available through their Data Archiving and Networked Services (DANS). This platform aims to make digital research data and related outputs findable, accessible, interoperable and reusable. All historical BBG files are available through this platform free of charge.

The land use categories used in the analyses (supplementary table 13) were based on the original classification of the BBG data (supplementary table 14). The categories were formed based on their potential relevance for walking and cycling. The spatial context of the cities included in the cohorts was taken into consideration in the selection of the land use categories. For example, the GLOBE cohort includes the city of Eindhoven and its more suburban surrounding areas. Therefore, agricultural areas were included as they might be relevant for cycling. The blue spaces category contains recreational water, such as canals and small lakes, which is especially relevant for the city of Amsterdam (LASA cohort).

**Supplementary table 13: Land use categories based on the land use classification of the BBG dataset**

| Land use categories | Corresponding BBG classifications |
| --- | --- |
| 1. Infrastructure | 10-12: Railway areas, road traffic areas, airports |
| 2. Residential areas | 20: Residential areas |
| 3. Retail areas | 21: Retail areas |
| 4. Public, social, and cultural facilities | 22-23: Public facility areas, social-cultural facility areas |
| 5. Other commercial areas | 24: Business areas |
| 6: Green facilities | 32, 41-44: Cemeteries, sports areas, allotment gardens, recreational areas, extended stay recreational areas |
| 7: Urban green spaces | 40: Parks |
| 8: Agricultural areas | 50-51: Greenhouses, general agricultural areas |
| 9: Forests and other natural areas | 60-62: Forests, open terrain (dry), open terrein (mixed) |
| 10: Blue spaces | 70-83: Lakes: IJsselmeer and Markermeer, closed estuaries, rivers: Rhine and Maas, border lakes, water reservoirs, recreational backwaters, water used for mineral extraction, sludge fields, other backwaters |
| 11: Other | 30-31, 33-35: Dumping grounds, junkyards, quarries, building sites, other |

**Supplementary table 14: Complete land use classification as translated by the authors**

| Main Group | Category | Lower Bounds (hectares) | Description |
| --- | --- | --- | --- |
| 1. Traffic areas |  |  |  |
|  | 10 | None | Railway areas |
|  | 11 | None | Road traffic areas |
|  | 12 | 1 | Airports |
| 2. Built environment |  |  |  |
|  | 20 | 1 | Residential areas |
|  | 21 | 1 | Retail areas |
|  | 22 | 1 | Public facility areas |
|  | 23 | 1 | Social-cultural facility areas |
|  | 24 | 1 | Business areas |
| 3. Semi-built areas | | | |
|  | 30 | 1 | Dumping grounds |
|  | 31 | 0.1 | Junkyards |
|  | 32 | 0.1 | Cemeteries |
|  | 33 | 0.5 | Quarries |
|  | 34 | 1 | Building sites |
|  | 35 | 1 | Other |
| 4. Recreational areas | | | |
|  | 40 | 1 | Parks |
|  | 41 | 0.5 | Sports areas |
|  | 42 | 0.1 | Allotment gardens |
|  | 43 | 1 | Recreational areas |
|  | 44 | 1 | Extended stay recreational areas |
| 5. Agricultural areas | | | |
|  | 50 | 1 | Greenhouses |
|  | 51 | 1 | General agricultural areas |
| 6. Forests and natural terrain | | | |
|  | 60 | 1 | Forests |
|  | 61 | 1 | Open terrain (dry) |
|  | 62 | 1 | Open terrein (mixed) |
| 7. Backwaters | | | |
|  | 70 | - | Lakes: IJsselmeer and Markermeer |
|  | 71 | - | Closed estuaries |
|  | 72 | - | Rivers: Rhine and Maas |
|  | 73 | - | Border lakes |
|  | 74 | 1 | Water reservoirs |
|  | 75 | 1 | Recreational backwaters |
|  | 76 | 1 | Water used for mineral extraction |
|  | 77 | 1 | Sludge fields |
|  | 78 | 1 | Other backwaters |
| 8. Open waters | | | |
|  | 80 | - | Specific open waters: Waddenzee, Eems, Dollard |
|  | 81 | - | Specific open waters: Oosterschelde |
|  | 82 | - | Specific open waters: Westerschelde |
|  | 83 | - | North Sea |
| 9. Borders | | | |
|  | 90 | - | Country borders |

# **SUPPLEMENTARY FILE 3**

**MORE INFORMATION ON THE RANDOM EFFECTS WITHIN-BETWEEN MODEL**

The random effects within-between model (REWB) goes by a variety of different names, such as the Mundlak model or hybrid model. The models used in our analyses are based on the work of Bell et al. (2019) and Lüdecke (2019).

Bell et al. (2019) describe a number of different REWBs with increasing degrees of complexity. They first present a general model (1) based on panel data example, where individuals $i$ (level 2) are measured on multiple occasions $t$ (level 1). This model is followed-up by a simplified model (2), that assumes homogeneous effects across level-2 entities. This is the model we have used for our analyses and which is presented in the main text.

Lüdecke (2019) has published a helpful article on how to apply the models presented by Bell et al. (2019) in statistical analyses using R. We used the lme4-package to specify our model parameters and to estimate the between and within effects based on the “simple” model as presented by Bell et al. (2019). We highly recommend Lüdecke’s guide for more information on how to specify REWB models in R.

**REFERENCES**

Bell A, Fairbrother M, Jones K. Fixed and random effects models: making an informed choice. Quality & Quantity. 2019; 53(2): p. 1051-74.

Lüdecke D, Fixed and Random Effects Models [Internet]; 2019. Available from: https://strengejacke.github.io/mixed-models-snippets/random-effects-within-between-effects-model.html
